# The Association Between Sexism, Self-Sexualization, and the Evaluation of Sexy Photos on Instagram

**DOI:** 10.3389/fpsyg.2021.716417

**Published:** 2021-08-25

**Authors:** Thomas Plieger, Olivia Groote, Rachel Hensky, Lea Hurtenbach, Sharon Sahler, Luise Thönes, Martin Reuter

**Affiliations:** Department of Psychology, University of Bonn, Bonn, Germany

**Keywords:** Instagram, objectification, self-sexualization, sexism, posting behavior, photo rating

## Abstract

Passive consumption of sexually objectifying content on social networking sites (SNS) has been shown to result in lowered body satisfaction and self-esteem, particularly in women. However, deliberate evaluations of sexually objectifying social media content are scarce. Furthermore, associations between self-objectification and active use of SNS in terms of posting behavior have not been shown so far. The present study asked *N* = 916 participants to rate 28 Instagram screenshots on three dimensions, namely, whether the given photos were perceived as sexually revealing, appropriate, and attractive. The ratings were related to sexist attitudes, enjoyment of sexualization, and posting behavior of the participants. Sexism was negatively associated with the perceived appropriateness of the presented Instagram photos in women. Furthermore, there were substantial correlations between appropriateness and attractiveness evaluations of the presented photos and the self-sexualizing posting behavior and enjoyment of sexualization of female users. Only inconsistent effects could be found in men. Greater appreciation of objectification seems to go along with self-objectifying behavior, which may be due to an altered perception of what can be considered as sexually revealing. Although effects seem to be smaller in men, future research should also focus on male enjoyment of sexualization, which unfortunately was not considered in the present study.

## Introduction

Social networking sites have gained a massive influence on the daily lives of billions of users at different levels. On the active side, they present a good platform for self-presentation from their mainly young users. On the passive side, social networking sites (SNS) are an informal and simple way to witness the allegedly day-to-day life of peers, celebrities, or influencers. Consequently, SNS have received increasing attention with respect to their associations with well-being, self-objectification, and other psychological variables of their users.

Sexual objectification, one specific form of objectification, occurs when the body and sexuality of a woman become the defining aspects of the description of the person as a whole. According to objectification theory, such experiences can result in women beginning to adopt an objectified third-party view on their physical selves; that is, if women are confronted with objectified views and evaluations of themselves and other women repeatedly, a stronger self-surveillance and a habitual self-monitoring of outer appearance become more likely. This is reinforced by the observation that people who are perceived as attractive are usually treated in a more favorable way (Fredrickson and Roberts, 1997). Thus, self-objectification happens when women accept being reduced to physical aspects of themselves and behave accordingly (e.g., greater body surveillance or self-presentation in a revealing manner). As the social media platform, Instagram is mainly characterized by visual stimuli, the present study will focus on a possible common form of (self-) objectification, namely, (self-) sexualization. Indeed, SNS are good outlets for young persons to present themselves in a self-objectifying, sexy, and desirable way (Zhao et al., 2008; Ringrose, 2011), as sexually objectifying content can often be found on social media. For example, it has been shown that a substantial share of Facebook profiles contains revealing or sexually suggestive pictures (Peluchette and Karl, 2009). Besides editing and uploading status updates and photos, users also spend large amounts of time consuming their newsfeeds (i.e., passive usage), which is substantially associated with variables concerning the recipients (Frison and Eggermont, 2017). For example, Facebook use and involvement have been associated with a more pronounced self-objectifying view of the body, a greater tendency toward enjoyment of sexualization, and more body image concerns (Vandenbosch and Eggermont, 2012; Meier and Gray, 2014; Manago et al., 2015). Instagram may even have a bigger impact on self-sexualization, as it is a SNS where users mainly communicate *via* visual stimuli (i.e., photographs). Accordingly, Instagram use has also repeatedly been associated with self-objectification (e.g., Fardouly et al., 2018; Feltman and Szymanski, 2018). Typically, SNS users tend to present their idealized selves. Furthermore, Feltman and Szymanski (2018) suggest that Instagram use might reinforce adherence to beauty standards, as users see that idealized pictures and pictures corresponding to ideals of beauty receive much attention in the form of likes and comments. Consequently, several studies have found that the association between SNS use and self-objectification is mediated by the internalization of beauty standards and the tendency to engage in social (upward) comparisons (e.g., Fardouly and Vartanian, 2015; Frison and Eggermont, 2017; Feltman and Szymanski, 2018).

At a more specific level, a recent study by Casale et al. (2019) let Instagram non-users watch attractive Instagram profiles over a 1-week period and observed greater body dissatisfaction and a stronger tendency toward appearance-focused self-evaluations in the experimental group, even if the effects did only reach significance in the female subsample. De Vries and Peter (2013) let young adult women create Facebook accounts after viewing sexually objectifying vs. neutral pictures, and found that women who were primed with sexualized stimuli created more self-objectifying Facebook pages. Taken together, many studies have investigated the influence of SNS use and sexually objectifying content on the body image, self-objectification, and self-esteem of users.

However, few studies have investigated how SNS users actually perceive photos they consume on Instagram or other SNS and which psychological constructs might predict the perception and evaluation of newsfeed content. For example, Daniels and Zurbriggen (2016a) let young women rate the owner of a female Facebook profile with either a sexualized or a non-sexualized profile photo. Except for the profile picture, the mock profile was exactly identical in both conditions. The results revealed lower physical attractiveness, social attractiveness, and competence ratings for the fictional Facebook profile owner with a sexualized profile photo. In a similar vein, female viewers evaluated other women from their peer group more negatively and as less intelligent when they believed that their photos had been manipulated with the use of filters or re-shaping (Vendemia and DeAndrea, 2018). In contrast, a study by Kleemans et al. (2018) showed higher attractiveness ratings for edited peer photos compared to unedited ones, although the participants were aware of which photos were manipulated and which were not.

Notwithstanding the fact that evidence for the deliberate evaluation of Instagram photos is scarce, it has also become apparent that the few studies that have investigated this topic so far have only tested female SNS users. It is safe to say that sexualizing and objectifying depictions of women have dominated both classic and social media in the past. Nonetheless, objectifications of men have increasingly appeared within the last decade. Consequently, some evidence suggests that objectifying depictions of men are related to self-objectification, internalization of beauty ideals, less positive affect, and body dissatisfaction in young men, too (e.g., Hobza and Rochlen, 2009; Rollero, 2013; Vandenbosch and Eggermont, 2013). Some other studies find only small or no effects of sexualizing media on body satisfaction or body consciousness of men. However, evidence suggests that men tend to increasingly invest more into their virtual appearance on SNS (e.g. Michaels et al., 2013; Manago et al., 2015).

As already mentioned, aside from a more differentiated view on gender, other psychological constructs may be important for understanding how self-objectifying social media content is perceived by the recipient. Karsay et al. (2018) state that socioeconomic status and gender role perceptions are understudied variables in the association between SNS use and self-objectification. Similarly, Feltman and Szymanski (2018) suggest the investigation of further variables such as traditional gender role adherence and attachment styles in the association between SNS use and self-objectification. Consequently, it would be interesting to relate these variables to the perception and evaluation of objectifying SNS content.

Traditional gender role stereotypes typically characterize women as weak, nurturing, passive, and less agentic. This traditional stereotypical view on women as being passive may be reflected in (self-) objectifying behavior and endorsement of (self-)objectification. Much of the adherence to traditional gender roles can be found in the theory of ambivalent sexism (e.g., Glick and Fiske, 1997, 2001, 2011), which covers hostile as well as benevolent aspects of gender stereotypes. Although Glick and Fiske have also hypothesized such ambivalence toward men, the theoretical concept mainly refers to sexism toward women and justification of the status quo in gender roles. The hostile sub-facet of sexism is defined by perceiving women as a threatening force trying to suppress men and challenge male dominance (e.g., by using their sexuality or complaining about structural gender discrimination). In contrast, benevolent sexism is characterized by the consideration of women as pure and fragile and, therefore, in need of male protection and care. Thus, ambivalent sexism is an interesting approach to explain how both negative and (in terms of traditional gender roles) positive attitudes toward women can represent two sides of the same coin: while hostile sexism emphasizes punishment, benevolent sexism emphasizes reward as part of the maintenance of traditional gender roles (Glick and Fiske, 2011). In fact, it could be shown that national average scores on both sexism subscales toward both sexes typically go along with gender inequality indices of the respective countries (Glick et al., 2004). Hence, sexism toward both women and men was positively associated with gender inequality. With respect to objectification, men scoring high on hostile sexism showed less neuronal activation in areas associated with mental state attribution when being presented with photos of sexualized female bodies (Cikara et al., 2011). Furthermore, male hostile sexism high-scorers perceived sexualized women as less agentic (i.e., as an object of action rather than as a person actively taking action) than non-sexists did in an implicit association test (Cikara et al., 2011). Similarly, Rollero (2013) has shown that the consumption of objectifying TV roles of females increases the endorsement of hostile sexist attitudes of men toward women while decreasing hostile sexism toward men. No effects were found in women. A recent study by Guizzo and Cadinu (2020) also presented different TV advertisements to a male sample and found that the presentation of sexualizing ads was related to greater hostile sexism, whereas the presentation of a sensitizing advertisement against the sexual objectification of women led to a greater endorsement of benevolent sexist attitudes. Associations between the consumption of sexually explicit material and sexist attitudes and the endorsement of traditional gender stereotypes have also been reported (e.g., Hald et al., 2013; Koletić, 2017). Despite these findings, there is, to the best of our knowledge, still no study linking sexist attitudes to the perception and evaluation of Instagram photos. Considering the large amounts of time people spend on SNS and their impact on daily life, research on how social media content may be associated with traditional gender role stereotypes and sexism is clearly needed. In contrast to classic media (e.g., TV), social media offers a possibility to not only consume but create sexualizing content. Therefore, drawing a link between the perception of consumed sexualizing content and a tendency for self-sexualizing behavior that, in turn, serves as sexualizing content for others again, is of high interest in order to understand the effects of SNS such as Instagram on their users.

Consequently, the current study aims to investigate whether sexist attitudes toward women relate to how content consumed *via* the SNS Instagram is perceived and evaluated by Instagram users and non-users, and how these evaluations are, in turn, associated with self-objectification. Specifically, we expected positive associations between the photo ratings (as attractive and appropriate) and enjoyment of sexualization. Furthermore, the vast majority of studies to date do not reveal any information about how the consumption of sexualized material may relate to the actual posting behavior of users (i.e., the behavioral outcome of self-objectification). Therefore, participants were asked about their own posting behavior in order to measure self-objectification more accurately, which represents a new methodological approach. In summary, the present study provides a possible model that explains the path from sexist attitudes to self-sexualization, which is mediated by the perception and evaluation of sexualized social media content. Paralleling the effects found for TV advertisements (Rollero, 2013; Guizzo and Cadinu, 2020), we also expected Instagram users to be more sexist and rate the presented photos as more appropriate and less revealing compared with non-users due to mere exposure to sexualizing stimuli.

## Methods

### Participants and Procedure

The online study was completed by 922 subjects. However, we decided to exclude six participants who self-identified themselves as diverse with respect to gender because this group was too small to produce reliable results. Therefore, the final sample comprised *N* = 916 (*n* = 680 female, *n* = 236 male) participants with a mean age of M = 27 (SD = 9.91). Of the participants, 662 (*n* = 500 female) reported to have an Instagram account, whereas 254 (*n* = 180 female) were Instagram non-users. The median of time spent online per day for those reporting to have an Instagram account was Md = 30 min (data only for 500 out of 662 Instagram users) and the median number of followers was Md = 178. Slightly more than half of the Instagram users (*n* = 350) had posted some kind of content (M = 1.18 posts and M = 3.38 stories) within the week before study participation, whereas *n* = 312 had not posted anything. One-third of the Instagram users had a public profile (i.e., any user can see uploaded content) and two-third of the users had a private profile (i.e., content can only be viewed by followers and other users have to send a request to follow the person).

The participants were recruited *via* advertisements posted to several social media networks and the distribution of a questionnaire link to friends and acquaintances of the experimenters. Participation in the study was anonymous. All participants gave informed consent by ticking a checkbox after they had received the study information. After agreeing to take part in this study, the participants were asked to provide some demographic information (e.g., age, sex, relationship status, educational level, political orientation, and life satisfaction), fill in several questionnaires, and rate Instagram photos (see below). At the end of the questionnaire, subjects had the opportunity to take part in a lottery for some Amazon vouchers.

### Instagram Stimuli

To measure the evaluation of other users portraying themselves online in a sexy way, the participants were presented with screenshots of Instagram photos.

First, we performed an exploratory picture search on Instagram by using several hashtags (e.g., #fashion, #instagood, #workout, #bikini, #fitnessgirl, #fitmen, and #fashiongirl). We only chose photos from influencers with 20,000–100,000 followers to minimize the chance that the participants would recognize the persons in the photos. This led to a photo set of 25 men and 25 women. In the pre-selection, we tried to parallelize photos of men and women (e.g., posing at the beach in swimwear or sitting on a weight bench in a gym in sportswear) so that we had 25 pairs of photos. The final selection was done after a small pre-study, in which we presented the 50 Instagram photos to 31 persons (*n* = 21 females; none of the subjects participated in the actual study). The photos had to be rated on the dimensions of posing behavior (i.e., whether or not the person is apparently intending to look sexy) and quantity of clothing. After a median split on both dimensions, we set up a 2 × 2 matrix and classified each photo in one of the resulting four groups. We only selected a pair for the actual study if the participants of the pre-study evaluated the man and the woman of the respective pair equally on both dimensions (i.e., classified them into the same group). Thus, the photos varied in their degree of sexualization in terms of clothing and posing (e.g., spread legs vs. sitting on a chair in a normal posture). Furthermore, the photos were revealing to different degrees, as some persons depicted in the photos had little clothing (e.g., a woman wearing a bikini or a man wearing tight sports pants and no shirt), whereas others showed less skin (e.g., a woman wearing jeans and a long sleeve shirt or a man with snowboard wear).

The final picture set was composed of 28 Instagram photos (14 pairs of pictures portraying a man or a woman, respectively) of attractive persons, which were shown to the participants in the study. The photos were presented in a randomized order. Captions, number of likes, and comments were blackened to avoid any confounding effects. Except for that, we used the original photos, as they had been posted on Instagram without actively controlling for or giving any hints about the extent to which the photos had been edited. The photos had to be rated on three dimensions on a seven-point Likert scale: (1) sexual revealingness of clothing (1 = not revealing at all, 7 = very revealing); (2) whether or not the photo is appropriate to be posted on Instagram (1 = very inappropriate, 7 = totally appropriate); (3) attractiveness of the photo (1 = not appealing at all, 7 = very appealing).

### Sexism

To measure sexism toward women, the Ambivalent Sexism Inventory (ASI; Glick and Fiske, 1996) was used. The ASI comprises 22 items which are divided into the two subscales: benevolent sexism (BS) and hostile sexism (HS). Sample items are “Most women interpret innocent remarks or acts as being sexist” (HS) and “Women should be cherished and protected by men” (BS). The items were answered on a six-point Likert scale (1 = strongly disagree; 6 = strongly agree). Internal consistencies measured by Cronbach's α were excellent for both subscales (HS: α = 0.93; BS: α = 0.89).

### Self-Sexualization

Self-sexualizing on Instagram was measured by asking the participants about their own posting behavior. Obviously, this is only of relevance to Instagram users, which is why we only asked subjects who reported owning an Instagram account. The participants were asked whether they would post a photo in underwear/swimwear, a photo in a sexually provocative pose, or a photo in underwear/swimwear showing them in a sexy pose on Instagram. All of the three items were answered on a five-point Likert scale ranging from “very unlikely” (1) to “very likely” (5).

Additionally, the participants were also asked whether they believed that their acceptance of sexual permissiveness has changed due to their Instagram usage (1 = less accepting; 4 = same as before; 7 = more accepting).

Furthermore, the female subsample also filled in the Enjoyment of Sexualization Scale (ESS; Liss et al., 2011). The ESS is composed of eight items (e.g., “It's important to me that men are attracted to me” and “I feel empowered, when I look beautiful”), which are answered on a six-point Likert scale (1 = disagree strongly; 6 = agree strongly). The scale proved to be reliable with Cronbach's α = 0.85 (only female subsample).

### Statistical Analyses

In order to test for any influences of the gender of participants on sexism, self-sexualization, and the evaluations of the presented Instagram screenshots, we conducted ANOVAs. Influences of age and sexual orientation were also tested *a priori* by correlational analyses. The associations between sexism, self-sexualization, and photo ratings were tested by partial correlations (see below). These analyses were calculated using SPSS 26 (IBM, Armonk, NY, USA). Furthermore, we performed structural equation modeling to conduct the path analyses with the use of the statistics program LISREL (8.8).

Data of the present study have been made available at https://osf.io/2xw9v/ for full transparency.

## Results

### Control Analyses

**Figure 1:**
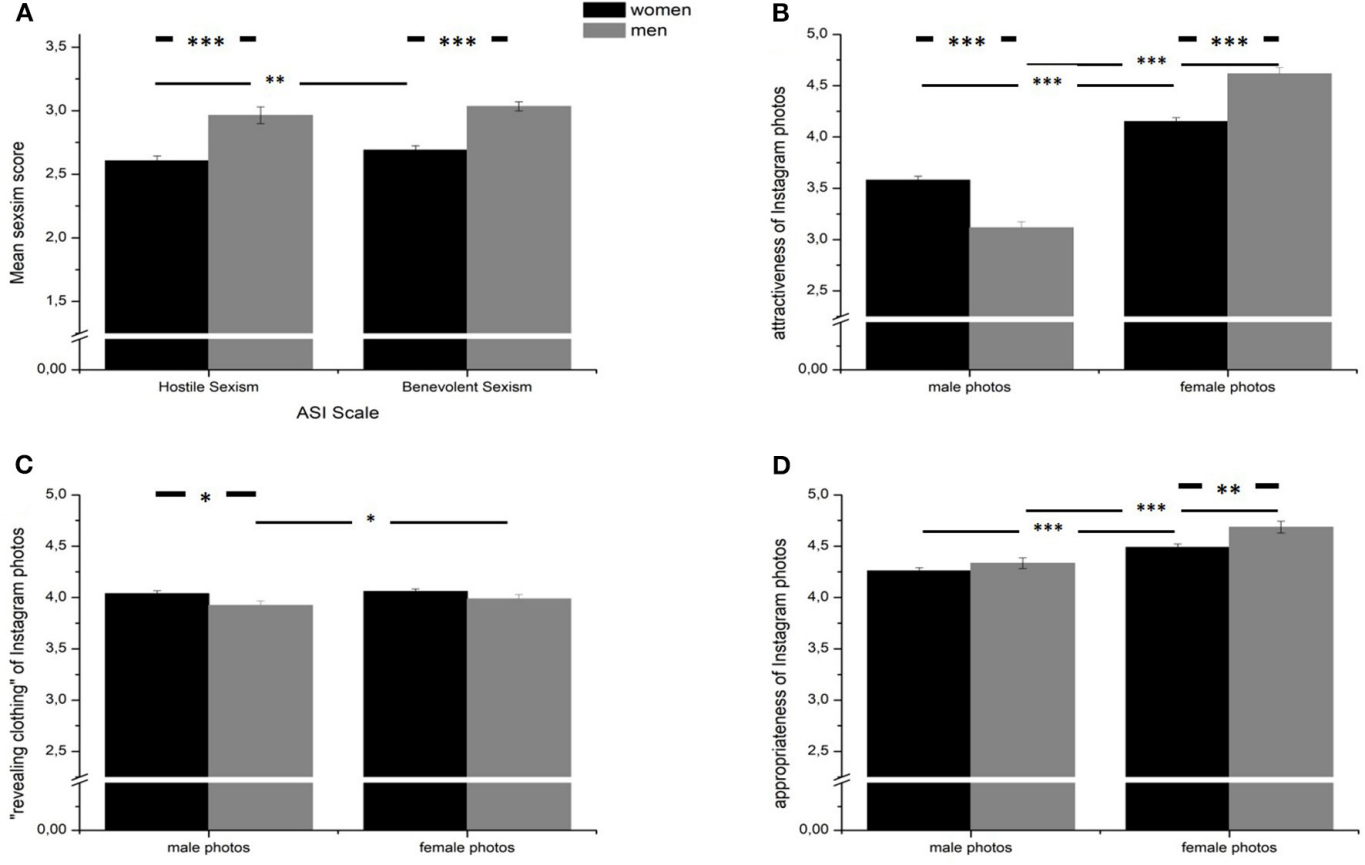
Mean differences between women (black) and men (gray) in **(A)** hostile and benevolent sexism and the three rating dimensions **(B)** attractiveness, **(C)** revealingness, and **(D)** appropriateness of the presented Instagram photos. *p < 0.05, **p < 0.01, ***p < 0.001.

First, we tested for sex differences in all variables under consideration (see Table 1 and Figure 1). Men scored higher in both HS (*F*_1, 914_ = 22.47, *p* < 0.001, η^2^ = 0.024) and BS (*F*_1, 914_ = 24.88, *p* < 0.001, η^2^ = 0.026) than women (see Figure 1A). The sexism scales correlated substantially in both sexes. Notably, the correlation in the female subsample (*r* = 0.624, *p* < 0.001) was significantly higher than in the male subsample (*r* = 0.470, *p* < 0.001) (*z* = 2.92, *p* = 0.004). Furthermore, men and women differed in the attractiveness ratings of the presented male (*F*_1, 914_ = 45.01, *p* < 0.001, η^2^ = 0.047) and female (*F*_1, 914_ = 41.01, *p* < 0.001, η^2^ = 0.043) screenshots (see Figure 1B). Thus, women found the male photos more attractive, whereas men rated the female photos as more attractive. In contrast, differences in the perception of revealing clothing of both male photos (*F*_1, 914_ = 5.34, *p* =0.021, η^2^ = 0.006) and female photos (*F*_1, 914_ = 2.11, *p* = 0.147) were smaller. Similarly, differences in the appropriateness ratings for male photos (*F*_1, 914_ = 1.53, *p* = 0.216) and female photos (*F*_1, 914_ = 8.94, *p* = 0.003, η^2^ = 0.010) were less pronounced (see Figures 1C,D).

**Table 1:** Descriptive statistics for all variables in male and female users.

	**Total sample**	**Men**	**Women**
	***M (SD)***	***M (SD)***	***M(SD)***
Benevolent sexism	2.77 (0.92)	3.03 (0.95)^***^	2.69 (0.89)^***^
Hostile sexism	2.69 (1.01)	2.96 (1.05)^***^	2.61 (0.98)^***^
Photo ratings			
Revealing	4.03 (0.63)	3.96 (0.62)^*^	4.05 (0.64)^*^
Appropriate	4.41 (0.81)	4.51 (0.77)^*^	4.37 (0.82)^*^
Attractive	3.87 (0.81)	3.87 (0.76)	3.87 (0.82)
Post in underwear	1.84 (1.16)	1.91 (1.21)	1.82 (1.14)
Post in a sexy pose	1.70 (1.05)	1.55 (0.99)^*^	1.75 (1.07)^*^
Post in underwear in a sexy pose	1.32 (0.80)	1.43 (0.93)^*^	1.28 (0.75)^*^
Acceptance of sexual permissiveness	4.54 (1.1)	4.55 (1.06)	4.54 (1.12)
ESS	-	-	3.63 (0.88)

* *p < 0.05,*

*** *p < 0.001 indicate significant differences between the male and female subsample (no control variables); Means for the evaluations of the presented Instagram photos include female and male photos. Figures 1B–D differentiate between male photos and female photos*.

We also tested whether age and sexual orientation were associated with the Instagram ratings or the independent variables of sexism and enjoyment of sexualization. Age was significantly and negatively associated with the rating of revealing clothing (*r* = −0.243, *p* < 0.001) and, to a lesser extent, with the rating of appropriateness (*r* = −0.094, *p* = 0.004). Furthermore, there was a negative association with the ESS score (*r* = −0.215, *p* < 0.001). HS was positively related to age (*r* = 0.113, *p* = 0.001), whereas there was no association between age and BS (*p* = 0.317). This means that younger participants perceived the presented photos as less revealing and younger female participants also reported to enjoy sexualization a little more than older participants, whereas older participants tended to have more hostile sexist attitudes toward women.

Sexual orientation (measured on a Likert scale: 1 = only interested in same sex; 4 = equally interested in all sexes; 7 = only interested in opposite sex) was related to HS (*r* = 0.196, *p* < 0.001) and BS (*r* = 0.192, *p* < 0.001), indicating more pronounced sexist attitudes in heterosexual people. There were no associations with the ratings of the Instagram photos. However, when considering the sex of participants, there were some significant correlations with attractiveness ratings within the male subsample. As one would expect, sexual orientation was negatively correlated with the attractiveness of male Instagram photos (*r* = −0.314, *p* < 0.001) and positively correlated with the attractiveness of female Instagram models (*r* = 0.245, *p* < 0.001). Except for a negligible association with attractiveness of male photos (*r* = 0.092, *p* = 0.016), there were no correlations between sexual orientation and ratings in the female subsample.

Consequently, we ran the analyses separated by sex and considered age and sexual orientation as covariates.

### Evaluation of Instagram Photos and Sexism Toward Women

With respect to the male subsample, there were no significant associations between the ratings of the photos and sexism. A positive correlation between BS and the attractiveness ratings of female Instagram photos (*r* = 0.127, *p* = 0.052) and a negative correlation between HS and the attractiveness ratings of male Instagram photos (*r* = −0.122, *p* = 0.062) missed significance. Notably, these correlations reached significance (*p* < 0.01) when we did not control for sexual orientation, but only for age. Thus, sexual orientation, although only moderately associated with sexism, seems to play a role in the association between male sexism and the perception of Instagram content.

In the female subsample, we found some associations between sexist attitudes and the appropriateness ratings of the presented photos (see Table 2). Particularly, the perceived appropriateness of the presented Instagram posts of both sexes was negatively associated with both HS and BS, suggesting a more liberal attitude toward the photos in participants with low sexism scores. Within the female participants, sexual orientation had almost no influence on these associations (indicated by virtually the same correlations when not controlling for it).

**Table 2:** Partial correlations between Instagram photo ratings and ambivalent sexism in the female subsample.

**Photo ratings**	**Hostile sexism**	**Benevolent sexism**
	***r*** **(** ***p*** **)**	
Revealing clothing males	**–0.131 (0.001)**	−0.063 (0.100)
Revealing clothing females	−0.086 (0.025)	−048 (0.211)
Appropriateness males	**–0.127 (0.001)**	**–0.194 (<0.001)**
Appropriateness females	**–0.108 (0.005)**	**–0.138 (<0.001)**
Attractiveness males	0.055 (0.155)	0.038 (0.320)
Attractiveness females	0.076 (0.047)	0.092 (0.017)

*Control variables: age, sexual orientation; N = 680. Correlations with p ≤ 0.001 are printed in bold*.

### Evaluation of Instagram Photos and Self-Sexualization

Self-sexualizing posting behavior was not related to the photo evaluation at all in male Instagram users. There was only a weak correlation between the likelihood of posting a photo in swimwear or underwear with the perceived appropriateness of the female Instagram photos (*r* =0.166, *p* = 0.042). With respect to the belief that Instagram has changed the acceptance one has for sexualizing media, there were positive correlations with the attractiveness (*r* = 0.196, *p* = 0.013) and appropriateness (*r* = 0.221, *p* = 0.005) ratings of female Instagram screenshots.

In women using Instagram, self-sexualizing posting behavior was significantly associated with all three evaluation dimensions. Women who self-reported that they would likely post a self-sexualizing photo on Instagram evaluated the degree of clothing significantly less revealing, perceived the presented photos as being more appropriate, and found the Instagram photos more attractive (see Table 3). All of these correlations were found to be independent of the gender of the Instagram model. The self-assessed acceptance of sexual permissiveness through the use of Instagram was also significantly related to appropriateness and attractiveness ratings. As described above, the female subsample also filled in the ESS, and women with a greater enjoyment of sexualization tended to rate the presented photos as more appropriate and attractive as well (see also Table 3).

**Table 3:** Partial correlations between Instagram photo ratings and the variables of self-sexualization in the female subsample.

**Photo ratings**	**Post in underwear**	**Post in a sexy pose**	**Post in underwear in a sexy pose**	**Acceptance of sexual permissiveness**	**ESS**
	***r*** **(** ***p*** **)**				
Revealing clothing M	**−0.167 (<0.001)**	**−0.166 (<0.001)**	**−0.253 (<0.001)**	0.011 (0.813)	−0.064 (0.240)^a^
Revealing clothing F	**−0.177 (<0.001)**	**−0.163 (<0.001)**	**−0.236 (<0.001)**	−0.019 (0.678)	−0.027 (0.486)^a^
Appropriateness M	**0.218 (<0.001)**	**0.191 (<0.001)**	0.131 (0.003)	**0.164 (<0.001)**	**0.122 (0.001)** ^**a**^
Appropriateness F	**0.249 (<0.001)**	**0.226 (<0.001)**	**0.159 (<0.001)**	**0.227 (<0.001)**	**0.157 (<0.001)** ^**a**^
Attractiveness M	0.105 (0.019)	0.132 (0.003)	0.105 (0.019)	0.116 (0.010)	**0.203 (<0.001)** ^**a**^
Attractiveness F	**0.181 (<0.001)**	**0.196 (<0.001)**	0.107 (0.017)	**0.159 (<0.001)**	**0.293 (<0.001)** ^**a**^

*Control variables: age, sexual orientation; M, male photo; F, female photo;*

a *N = 680 (N = 500 for all other correlations). Correlations with p ≤ 0.001 are printed in bold*.

### Sexism, Perception of Instagram Photos, and Self-Sexualization

After correlating ambivalent sexism with the perceptions participants had of the presented photos, and then correlating these perceptions with different indicators of self-sexualization, we tried to merge all of the variables into one model. In order to do so, we conducted an exploratory and data-driven path analysis using the structural equation modeling (SEM) tool LISREL (8.80). Notably, we only performed SEM for the female subsample and did not differentiate in the evaluations of female and male photos anymore to keep the model comprehensive.

**Figure 2:**
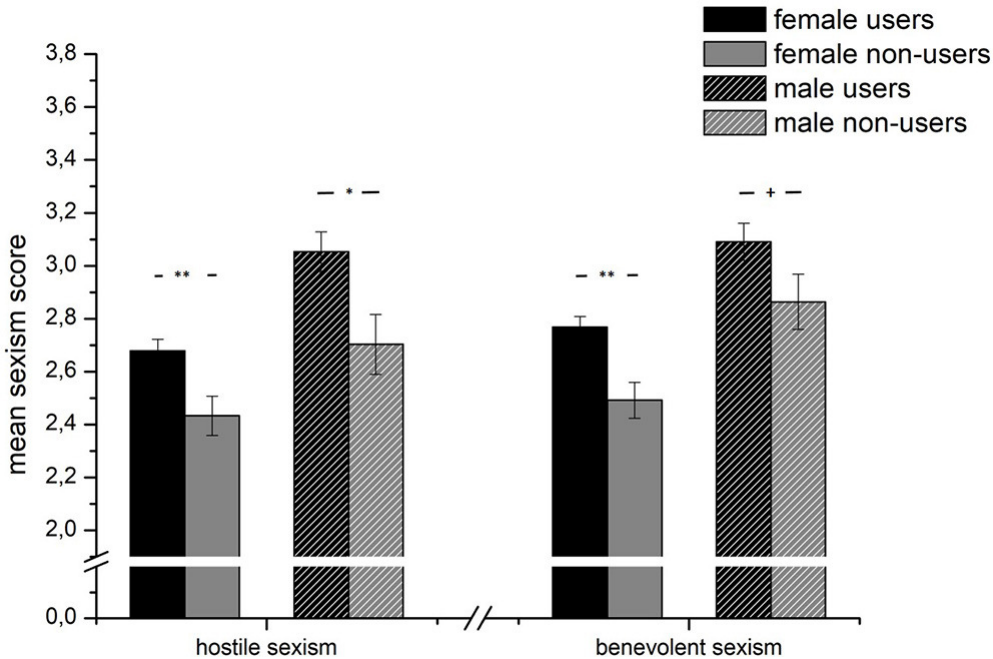
Mean differences between Instagram users (black) and non-users (gray) separated by sex for hostile and benevolent sexism measured by the Ambivalent Sexism Inventory (ASI). ^+^*p* < 0.1, *p < 0.05, **p < 0.01.

The resulting model showed a good fit (chi^2^ = 23.71, df = 21, *p* = 0.307; RMSEA = 0.014; CFI = 1) and included direct paths between sexism toward women and ESS, as well as the willingness to post sexy and revealing photos on a personal Instagram profile (see Figure 2). Moreover, sexism was associated with the evaluations of the presented photos as revealing and (in-)appropriate, which in turn were associated with the acceptance of sexual permissiveness and the willingness to post revealing photos. The perception of the presented photos as attractive was not associated with sexism, but showed significant paths to the enjoyment of sexualization and the intention to post revealing photos.

### Instagram Users vs. Non-users

**Figure 3:**
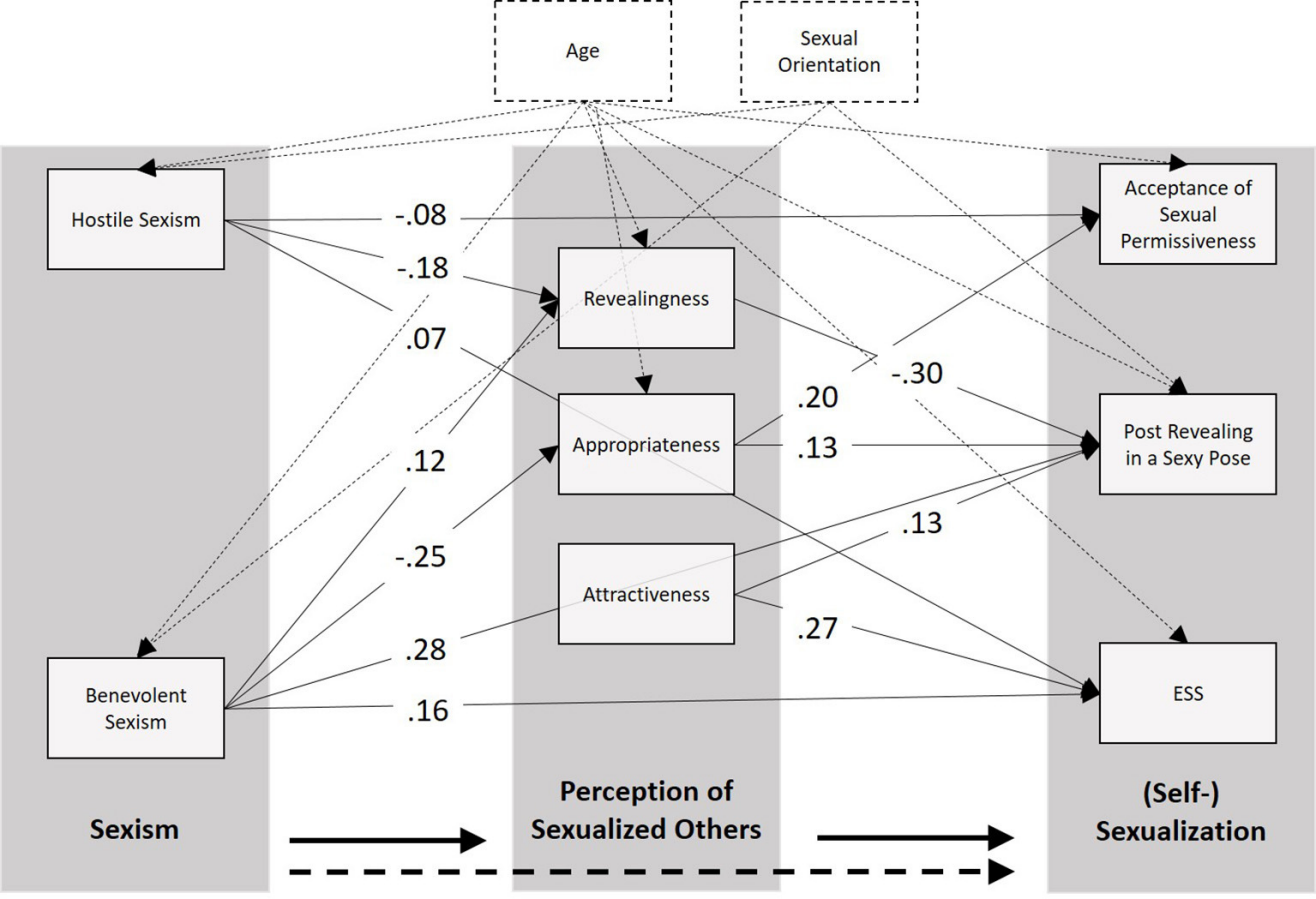
Final path model (chi^2^ = 23.71, df = 21, *p* = 0.307; RMSEA = 0.014) from ambivalent sexism (hostile and benevolent) *via* the mean evaluations of the presented Instagram photos (as revealing, appropriate, and attractive) to (self-) sexualizing behaviors and attitudes. All shown paths are significant at *p* < 0.05; for a better readability of the figure, the coefficients for the control variables age and sexual orientation are not depicted.

On a more general level, we also tested whether there were differences in sexism and the perception and evaluation of the presented Instagram photos between users and non-users. In fact, we observed both higher BS (*F*_1, 914_ = 13.16, *p* < 0.001, η^2^ = 0.014) and HS (*F*_1, 912_ = 12.24, *p* < 0.001, η^2^ = 0.013) scores in Instagram users compared to non-users (see Figure 3).

Furthermore, users perceived the presented photos of women as less revealing and more attractive. The presented photos of both sexes were rated as more appropriate by users compared to non-users (see Table 4). Moreover, female users scored higher in enjoyment of sexualization than non-users (*F*_1, 676_ = 9.34, *p* = 0.002, η^2^ = 0.014).

**Table 4:** Differences between Instagram users and non-users in the evaluation of the presented Instagram photos.

**Photo ratings**	**Instagram users**	**Instagram non-users**	**Ancova**
	***M (SD)***	***M(SD)***	***df: 1, 912***
Revealing clothing M	4.00 (0.66)	4.03 (0.68)	*F* = 9.09, *p* = 0.003, η^2^ = 0.010
Revealing clothing F	4.01 (0.66)	4.13 (0.66)	*F* = 15.47, *p* <0.001, η^2^ = 0.017
Appropriateness M	4.35 (0.79)	4.09 (0.79)	*F* = 15.05, *p* <0.001, η^2^ = 0.016
Appropriateness F	4.66 (0.84)	4.23 (0.87)	*F* = 38.58, *p* <0.001, η^2^ = 0.041
Attractiveness M	3.49 (0.92)	3.36 (0.98)	*F* = 3.98, *p* = 0.046, η^2^ = 0.004
Attractiveness F	4.35 (0.97)	4.05 (0.99)	*F* = 14.33, *p* <0.001, η^2^ = 0.015

*Control variables: age, sexual orientation; M, male photos; F, female photos; N = 916*.

## Discussion

The aim of the present study was to investigate how attractive Instagram photos of others are perceived by Instagram users and non-users. The participants evaluated a set of male and female Instagram screenshots with respect to three aspects, namely, how revealing the photos were, whether they found such photos appropriate to be posted on Instagram, and whether they found the photo attractive. The ratings were related to sexist attitudes and self-objectifying behavior of the participants.

The control analyses suggested substantial differences between men and women. Men were found to be more sexist toward women than women, which is in line with several other studies (e.g., Russell and Trigg, 2004; Ramiro-Sánchez et al., 2018). Similarly, a study by Brandt (2011) shows gender differences in samples of over 50 countries. Unfortunately, he did not differentiate between HS and BS, which may not be trivial; as Allen et al. (2009) also found higher HS scores in men, but no sex differences in BS at all. Notably, BS and HS showed a closer association in the female subsample than in the male subsample, which was also suggested by cross-cultural findings from Glick et al. (2000, 2004). Men and women also differed in their attractiveness ratings of the photos (see Figure 1B), which was clearly due to the mainly heterosexual sample. However, sexual orientation significantly contributed to sexist attitudes, in that heterosexual people of both sexes tended to be more sexist than homosexual participants. Thus, deviating from the traditional exclusive interest in persons of the opposite sex seems to go along with turning away from traditional, sexist stereotypes about women.

With respect to the Instagram photos, sexist attitudes were not related to any of the rating dimensions in men when we controlled for sexual orientation. This is quite surprising, as sexism represents the perpetuation of traditional gender stereotypes. Thus, we expected that sexist men would evaluate the objectified depiction of women as more appropriate than non-sexist men. In line with this assumption, Rollero (2013) and Guizzo and Cadinu (2020) showed an association between the presentation of sexually objectified women and hostile sexism. The contrary findings of this article underline that it may indeed be useful not only to present social media content to participants, but to also ask for deliberate evaluations of the presented pictures, as this may give additional insights into how social media platforms affect their users. Kleemans et al. (2018) recently showed that photos that are rated most positive and attractive have the strongest effect on body dissatisfaction. Thus, the conscious perception of Instagram content may deviate from its actual impact on its recipients. Another possible explanation for the findings of the authors lies within the theoretical framework: Hostile sexism also implies the belief that women use their physical attractiveness to gain power over men. Hence, the null-findings might be due to the opposing effects of endorsing the sexual objectification of women on the one hand, and condemning the instrumental use of attractiveness on the other. This contradiction was recently labeled as the “Madonna-Whore Dichotomy” by Bareket et al. (2018). In the female subsample, the appropriateness ratings of both male and female photos were negatively associated with BS and HS. Fittingly, Chapleau et al. (2007) found positive associations between ambivalent sexism and Rape Myth Acceptance (e.g., the woman provoked a sexual offense by her behavior or dressing style) in a predominantly female sample. Unfortunately, the study did not report its results separated by gender. However, similar results were found for sexual harassment in purely female samples (Lonsway et al., 2008; del Carmen Herrera et al., 2017). Accordingly, female sexism toward their own gender is apparently linked to the endorsement of allegedly appropriate behavior of women. Notably, sexism was only inconsistently related to the perceived degree of revealing clothing or attractiveness ratings. Apparently, sexism has nothing to do with the liking of a photo or the perception of permissiveness or revealing clothing, but is associated with the moral judgment of it.

Despite gender differences in the association between sexism and the Instagram photo ratings, Instagram users of both sexes showed stronger benevolent and hostile sexist attitudes toward women than Instagram non-users. Thus, not only do sexually objectifying TV advertisements or pornographic material (Koletić, 2017; Guizzo and Cadinu, 2020) promote sexist attitudes and the adherence to traditional gender stereotypes but also the mere use of Instagram seems to do so as well, which may in turn lead to a more objectified and sexualized view of women.

The variables measuring self-presentation on social networking sites were not substantially associated with the perception of the photos in men. In contrast, there were substantial associations between self-sexualizing posting behavior and the evaluation of the presented Instagram photos on all rating dimensions (i.e., revealing clothing, perceived appropriateness, and attractiveness) in the female subsample. The positive correlations between self-objectifying posting behavior and the perceived appropriateness of other people's posts are not surprising at all, and reflect the consistency of female participants in what they believe is admissible for presentation to the virtual community on Instagram. Consequently, women reporting self-sexualizing posting behavior also find other posts by women on Instagram more attractive. Interestingly, posting behavior correlated negatively with the perception of the presented photos as revealing. Hence, women who reported sharing self-sexualizing photos of themselves on social media platforms might do so because they do not actually perceive them as being too revealing or sexually permissive. This may be due to a habituation to sexually revealing social media content, with this explanation being supported by the finding that participants with an Instagram account rated the photos as more appropriate and perceived the female photos as less revealing. Alternatively, the more liberal ratings of the photos might also be a *post-hoc* justification of self-objectifying posting behavior. Either way, the mere (active or passive) use of Instagram seems to be associated with the perception of objectified depictions of others. These results are in line with studies that found a positive association between Instagram use and self-objectification (Fardouly et al., 2018; Feltman and Szymanski, 2018). In fact, the female Instagram users in this study also showed a greater enjoyment of sexualization than non-users. Nonetheless, due to the cross-sectional nature of the present study, the direction of this effect remains unclear. Although less likely, it may also be possible that people who are genuinely more accepting of sexualization in social media choose to use Instagram either actively (e.g., by self-sexualizing behavior) or passively (e.g., by consuming sexualizing content). However, the self-assessed increased acceptance of sexual permissiveness through the use of Instagram did not correlate with the assessed degree of revealing clothing, which speaks against habituation as an explanation for the found associations. Time spent on Instagram was not associated with posting behavior or the picture ratings either (data not shown), further questioning the likelihood of a habituation effect being an explanation for the different (i.e., more liberal) perception of sexualized stimuli. The effects might also be promoted by trait-like characteristics rather than by habituation, as enjoyment of sexualization was positively associated with the appropriateness and attractiveness ratings. Thus, women who like to feel sexy and like to be complimented for appearing in a sexy way appreciated sexy photos of others and did not judge such photos as too permissive. Consistently, the ESS scores correlated positively with self-sexualizing posting behavior (all *p*'s < 0.001), which underlines that enjoyment of sexualization may be an underlying mechanism for personal social media usage behavior and the perception of (self-)objectifying social media content of other people. However, it is conceivable that Instagram use and enjoyment of sexualization reinforce each other; making an assumption of unidirectional causality is hardly justifiable without any longitudinal data.

The different correlational patterns between sexes give support to the notion that effects of social media use seem to be less pronounced in men as compared to women. With respect to effects of social media on self-objectification, body shame, and body dissatisfaction, other workgroups have already suggested the same findings (e.g., Michaels et al., 2013; Manago et al., 2015). Similarly, Fox and Rooney (2015) only find very small associations between male self-objectification and selfie-posting and photo editing. In contrast, the meta-analysis by Karsay et al. (2018) reports no sex effects in the association between Facebook involvement and objectified body consciousness. Taken together, more research in men is needed to get a better understanding of social media exposure effects on self-objectification.

### Limitations and Future Research

Some aspects of this study deserve critical consideration. First of all, although we propose a path model, we cannot really tell how the causal relationships between sexism, the perception of Instagram stimuli, Instagram use, and self-objectification are because of the cross-sectional design. Furthermore, the model is driven by the results and fitted to the data. Therefore, future longitudinal studies can help to get a more accurate impression of the direction of the found effects. Nonetheless, the model provides a possible explanation for how social networks may become self-perpetuating systems in terms of (self-)objectification.

Another shortcoming of this study relates to the instruments. We decided to ask for the enjoyment of sexualization only in the female sub-sample. Undoubtedly, some information about the enjoyment of sexualization in men would also have been interesting. However, the ESS has originally been designed for women and is almost exclusively used in female samples, which is why we simply did not think about the option of also handing it to the male participants. Similarly, we decided to ask for sexism toward women only. A small sub-sample of about 150 participants also filled in the Ambivalence toward Men Inventory (AMI; Glick and Fiske, 1999). The AMI was dropped for the sake of time and economic reasons in order to decrease the dropout rate. As sexism toward women is still the much more salient and common form of gender inequality and objectification in classic and social media, we believed that the ASI was of higher relevance. However, sexism toward men is an important issue that should not be neglected in the future, as it is becoming increasingly important in the context of (self-)sexualization of men in social media. The last point of criticism of the design in this study concerns the items measuring the posting behavior of participants. The first question was “Would you post a photo of you in swim- or underwear on Instagram?” This item produced very little variance in response behavior. It might have been better to ask for the likelihood of posting a photo in swimwear or underwear separately, as this can make a difference for many people (Daniels and Zurbriggen, 2016b). While photos of people at the beach or at the pool are quite common on SNS, people rarely show themselves in underwear unless they are professional influencers. Thus, it is likely that—although equally revealing in an objective sense—photos of these two categories are not perceived as equally sexually permissive. Furthermore, it would have been more informative to scan the actual Instagram profiles for self-sexualizing posts or to let the participants count, e.g., pictures of themselves in swimwear on their account instead of asking them whether or not they would be willing to post such a photo. This would have allowed insight into the actual behavior of the participants rather than intentions to behave in a certain way. However, this would have made data acquisition much more complex and could have led to a higher dropout rate because the participants, at least those with private accounts, would have been forced to share their accounts with us. Nonetheless, future studies could benefit from a more direct measure of self-sexualizing behavior.

## Conclusion

In conclusion, the present study shows associations between the perception of Instagram content, sexism, and the posting behavior of users. Notably, these associations were only consistently found in the female subsample. Sexist women tended to rate Instagram photos of both men and women as less appropriate. With respect to self-sexualization, women who reported a greater likelihood of posting self-sexualizing photos on Instagram found the presented Instagram screenshots less revealing and more appropriate. This finding suggests that women who act more liberally also judge other persons more liberally, and that both engaging in and evaluating permissive Instagram posting behavior may be a result of perceiving this behavior simply as less permissive. Furthermore, women with a greater enjoyment of sexualization found the presented pictures more appropriate and more attractive, which also hints toward a substantial coherence between the liking of one's own sexy appearance and the liking of photos where other persons are depicted in a sexy way.

The present study also aimed to learn more about the effects of social media usage in men. As other studies have already suggested, the effects seem to be smaller (if existent at all) in men. However, future studies should consider sexism toward men and enjoyment of sexualization not only in women but also in men.

## Data Availability Statement

The datasets presented in this study can be found in online repositories. The names of the repository/repositories and accession number(s) can be found below: https://osf.io/2xw9v/.

## Ethics Statement

Ethical review and approval was not required for the study on human participants in accordance with the local legislation and institutional requirements. The patients/participants provided their written informed consent to participate in this study.

## Author Contributions

TP and MR: study design and writing of the manuscript. OG: study design and data acquisition. RH, LH, SS, and LT: data acquisition. All authors contributed to the article and approved the submitted version.

## Conflict of Interest

The authors declare that the research was conducted in the absence of any commercial or financial relationships that could be construed as a potential conflict of interest.

## Publisher's Note

All claims expressed in this article are solely those of the authors and do not necessarily represent those of their affiliated organizations, or those of the publisher, the editors and the reviewers. Any product that may be evaluated in this article, or claim that may be made by its manufacturer, is not guaranteed or endorsed by the publisher.
